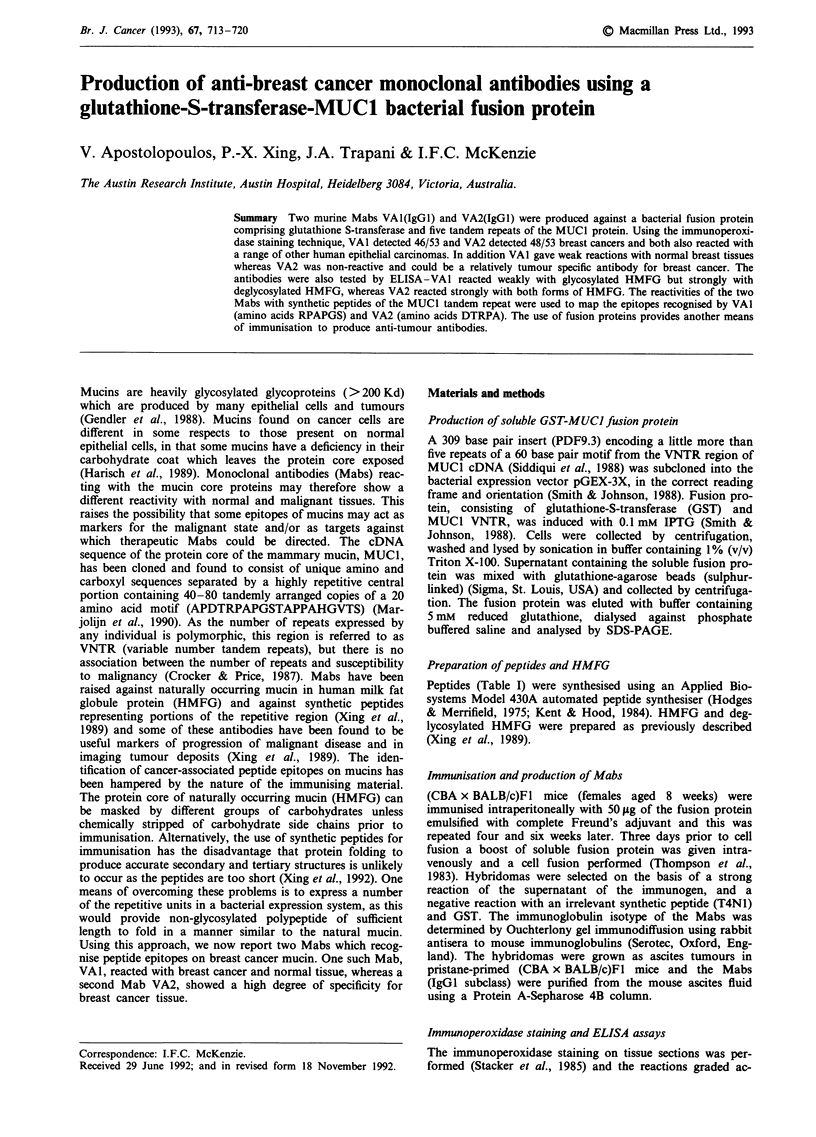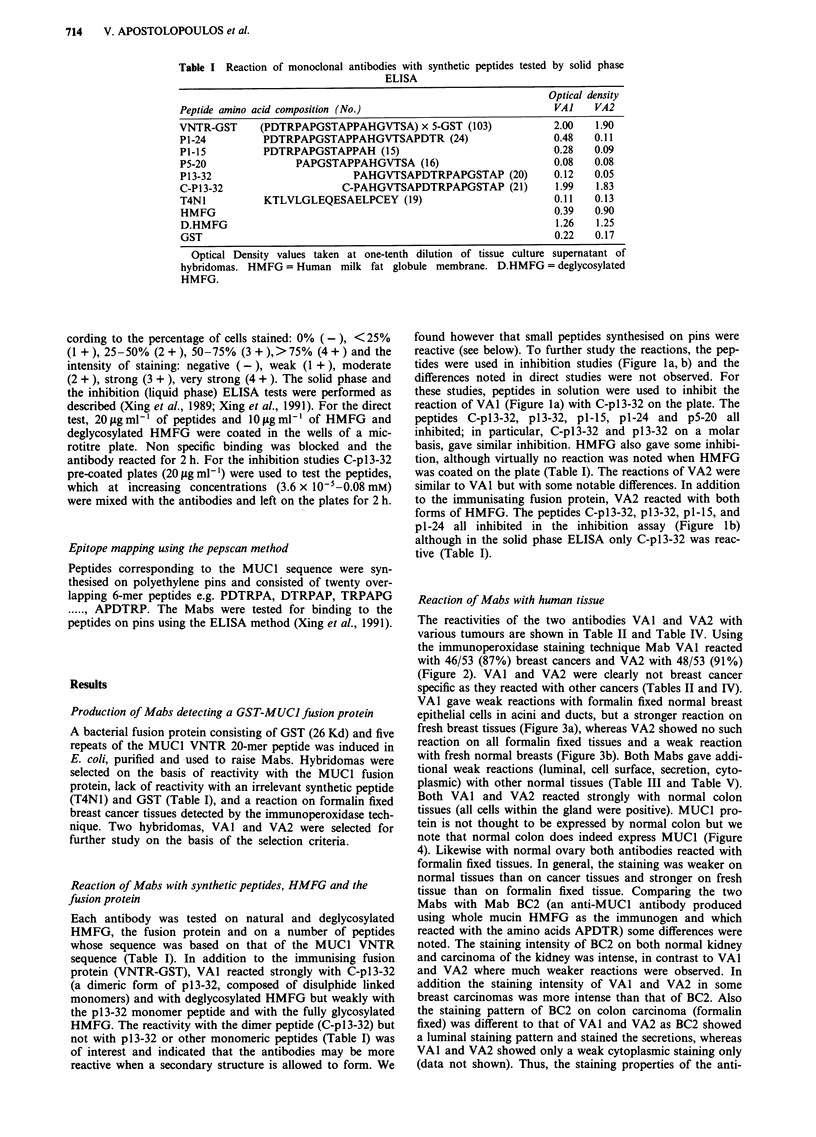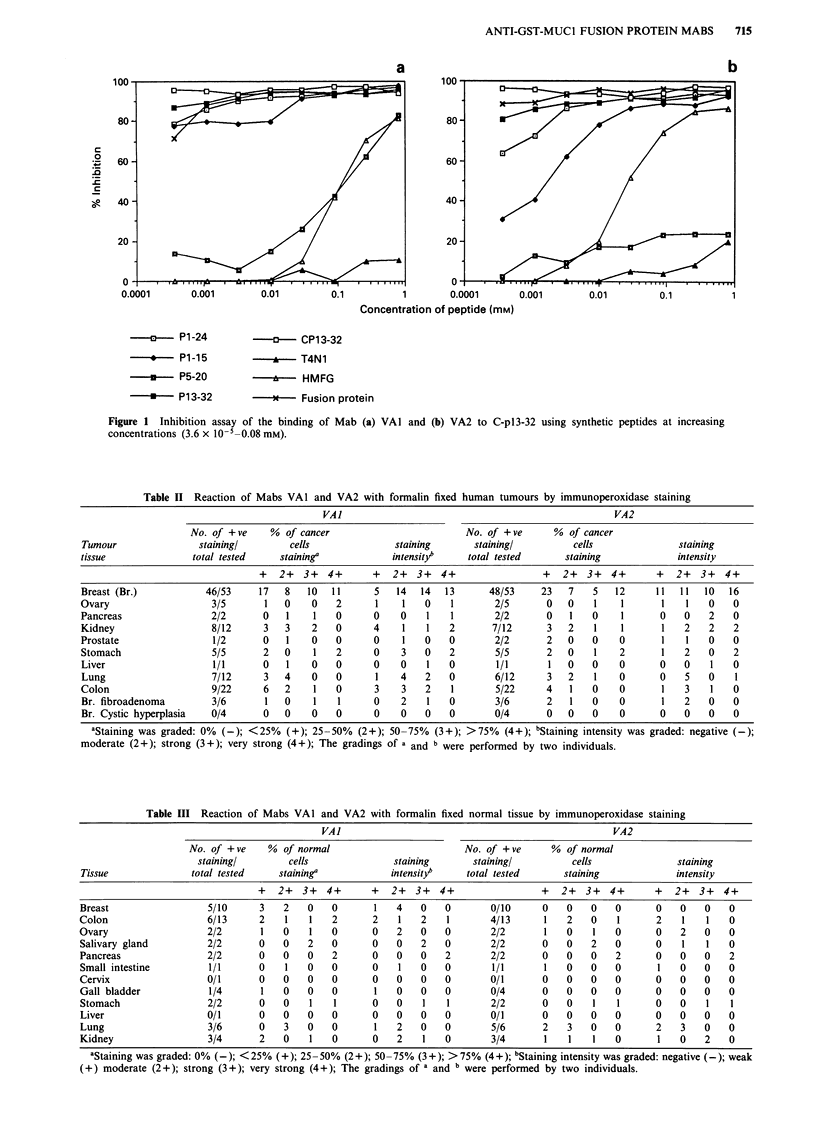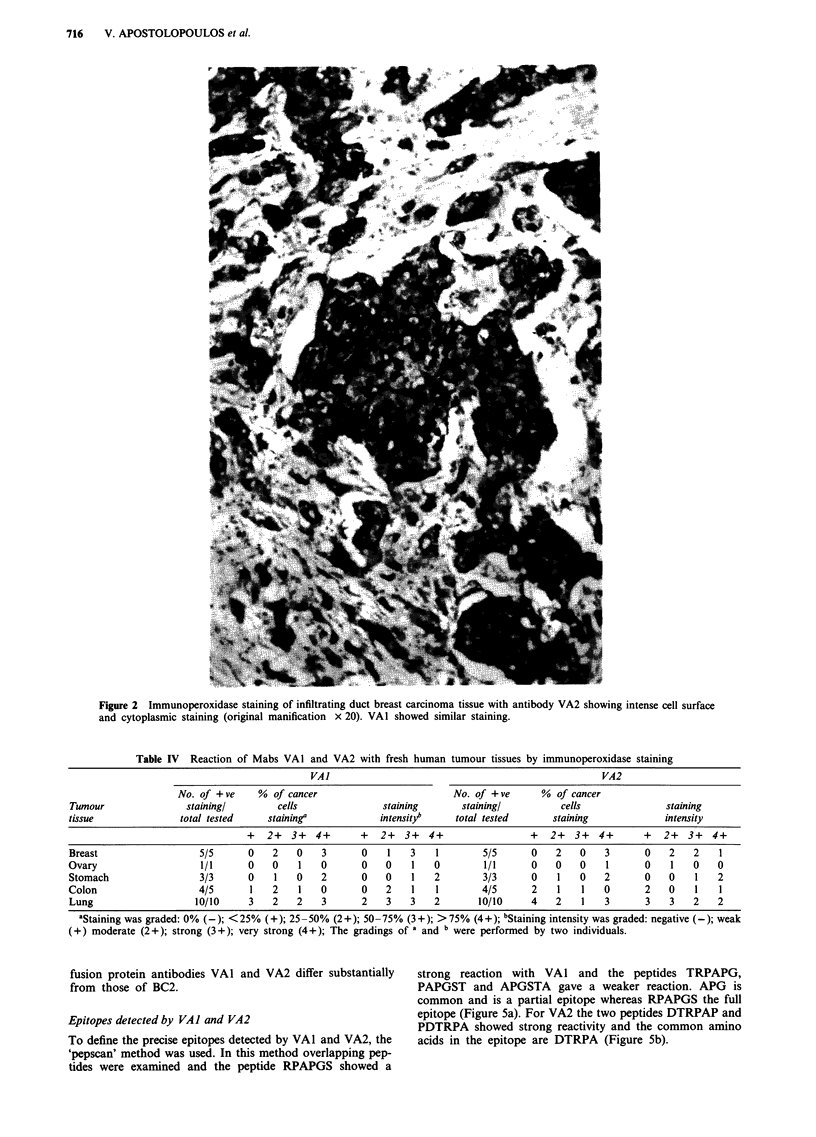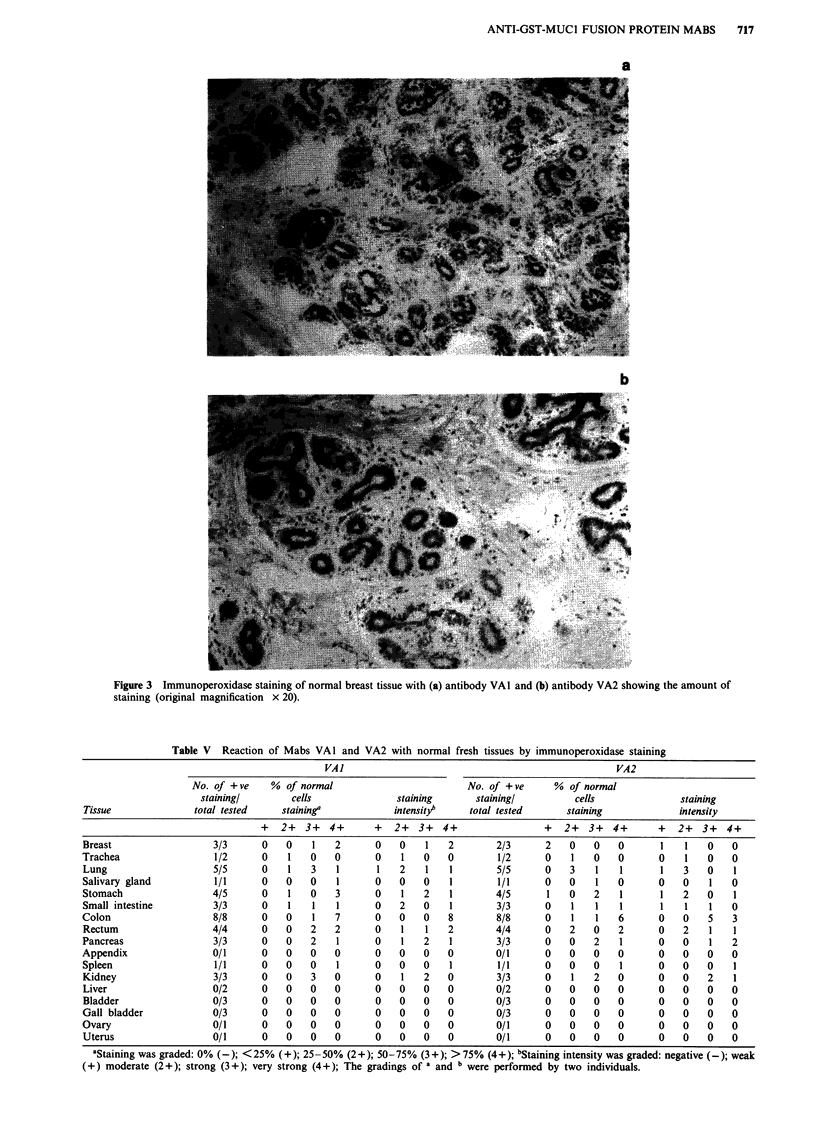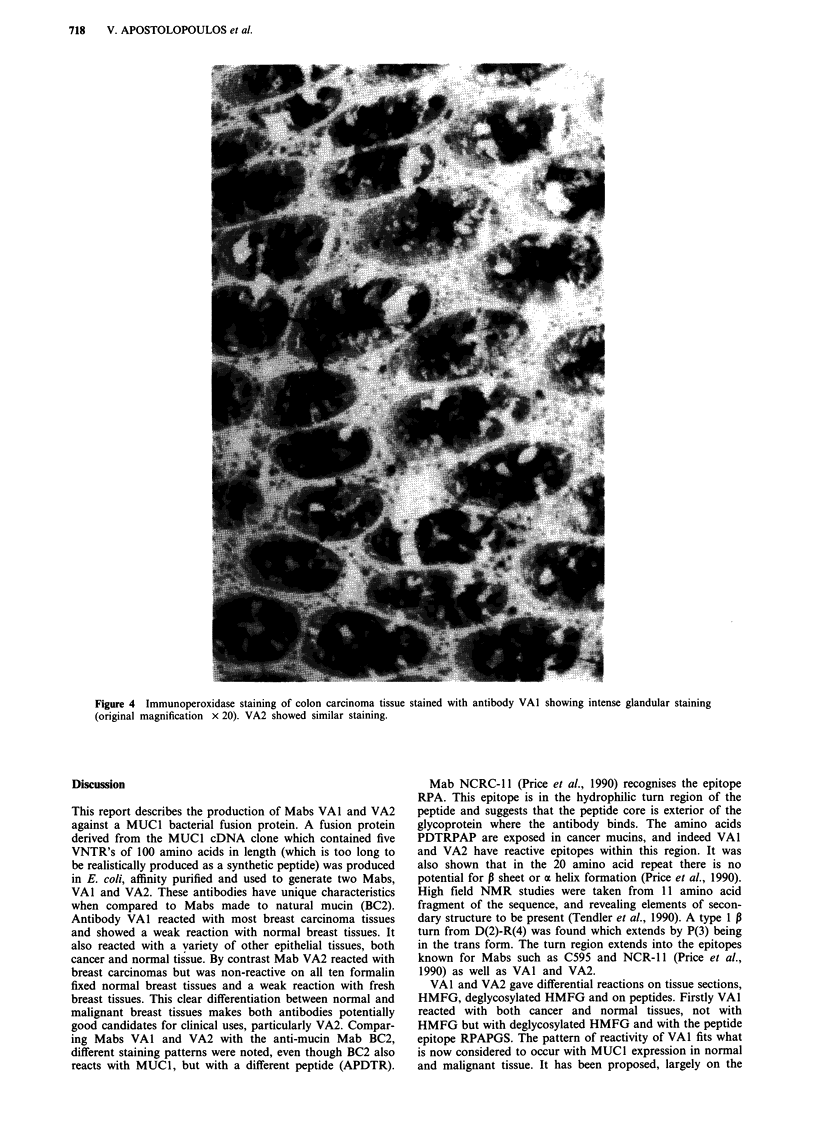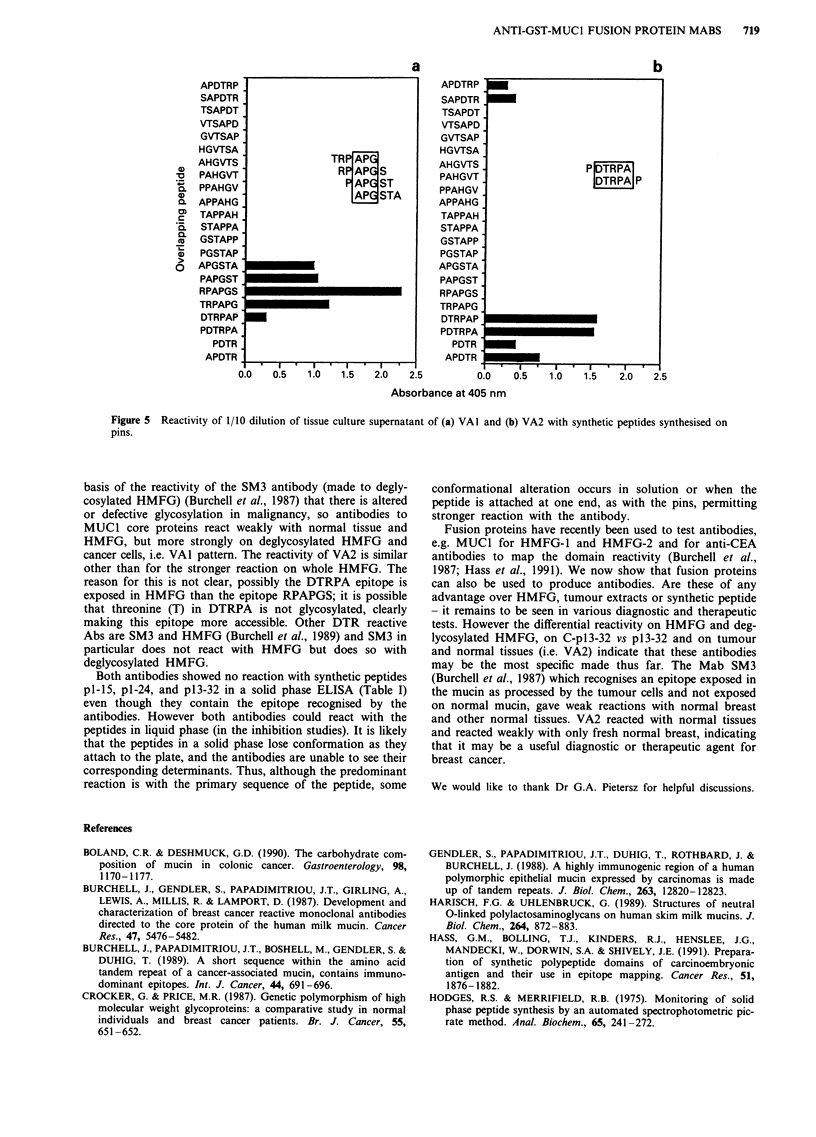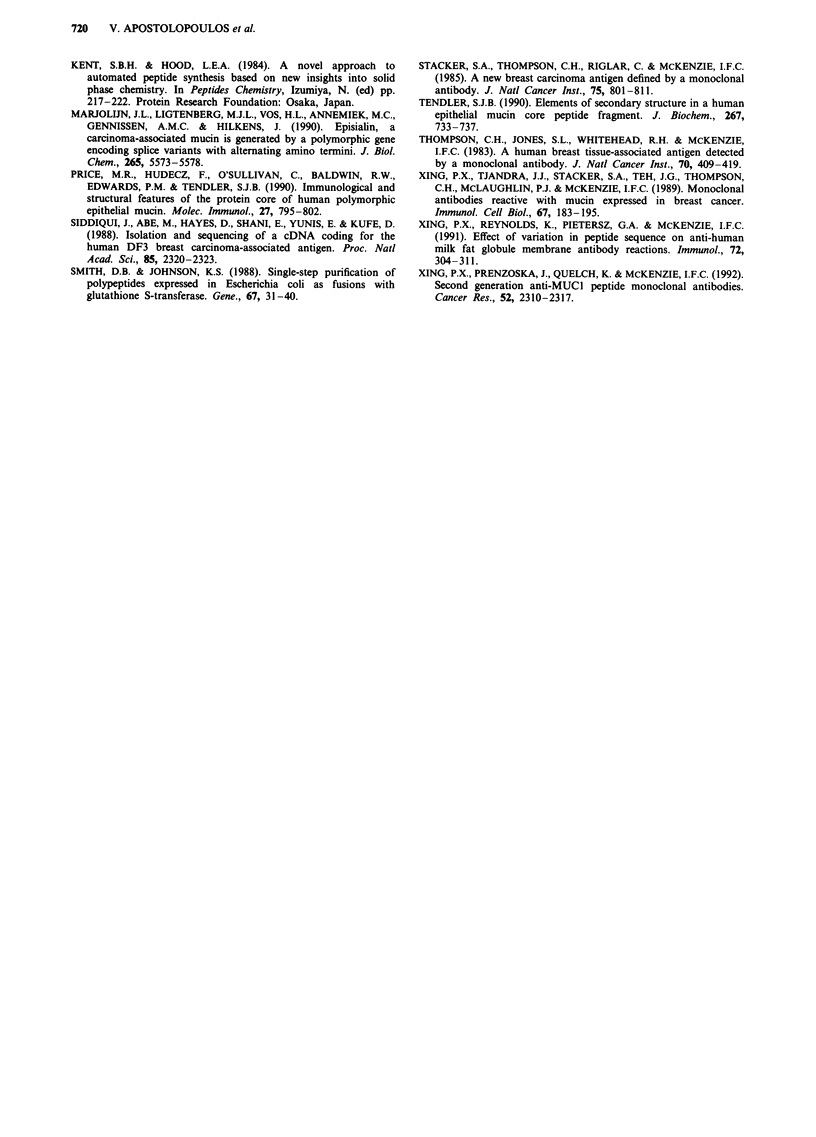# Production of anti-breast cancer monoclonal antibodies using a glutathione-S-transferase-MUC1 bacterial fusion protein.

**DOI:** 10.1038/bjc.1993.131

**Published:** 1993-04

**Authors:** V. Apostolopoulos, P. X. Xing, J. A. Trapani, I. F. McKenzie

**Affiliations:** Austin Research Institute, Austin Hospital, Heidelberg, Victoria, Australia.

## Abstract

**Images:**


Br. J. Cancer (1993), 67, 713-720

© Macmillan Press Ltd., 1993

### Production of anti-breast cancer monoclonal antibodies using a glutathione-S-transferase-MUC1 bacterial fusion protein

V. Apostolopoulos, P.-X. Xing, J.A. Trapani & I.F.C. McKenzie

*The Austin Research Institute, Austin Hospital, Heidelberg 3084, Victoria, Australia.*

**Summary** Two murine Mabs VA1(IgG1) and VA2(IgG1) were produced against a bacterial fusion protein comprising glutathione S-transferase and five tandem repeats of the MUC1 protein. Using the immunoperoxidase staining technique, VA1 detected 46/53 and VA2 detected 48/53 breast cancers and both also reacted with a range of other human epithelial carcinomas. In addition VA1 gave weak reactions with normal breast tissues whereas VA2 was non-reactive and could be a relatively tumour specific antibody for breast cancer. The antibodies were also tested by ELISA-VA1 reacted weakly with glycosylated HMFG but strongly with deglycosylated HMFG, whereas VA2 reacted strongly with both forms of HMFG. The reactivities of the two Mabs with synthetic peptides of the MUC1 tandem repeat were used to map the epitopes recognised by VA1 (amino acids RPAPGS) and VA2 (amino acids DTRPA). The use of fusion proteins provides another means of immunisation to produce anti-tumour antibodies.

Mucins are heavily glycosylated glycoproteins (>200 Kd) which are produced by many epithelial cells and tumours (Gendler *et al.*, 1988). Mucins found on cancer cells are different in some respects to those present on normal epithelial cells, in that some mucins have a deficiency in their carbohydrate coat which leaves the protein core exposed (Harisch *et al.*, 1989). Monoclonal antibodies (Mabs) reacting with the mucin core proteins may therefore show a different reactivity with normal and malignant tissues. This raises the possibility that some epitopes of mucins may act as markers for the malignant state and/or as targets against which therapeutic Mabs could be directed. The cDNA sequence of the protein core of the mammary mucin, MUC1, has been cloned and found to consist of unique amino and carboxyl sequences separated by a highly repetitive central portion containing 40-80 tandemly arranged copies of a 20 amino acid motif (APDTRPAPGSTAPPAHGVTS) (Marjolijn *et al.*, 1990). As the number of repeats expressed by any individual is polymorphic, this region is referred to as VNTR (variable number tandem repeats), but there is no association between the number of repeats and susceptibility to malignancy (Crocker & Price, 1987). Mabs have been raised against naturally occurring mucin in human milk fat globule protein (HMFG) and against synthetic peptides representing portions of the repetitive region (Xing *et al.*, 1989) and some of these antibodies have been found to be useful markers of progression of malignant disease and in imaging tumour deposits (Xing *et al.*, 1989). The identification of cancer-associated peptide epitopes on mucins has been hampered by the nature of the immunising material. The protein core of naturally occurring mucin (HMFG) can be masked by different groups of carbohydrates unless chemically stripped of carbohydrate side chains prior to immunisation. Alternatively, the use of synthetic peptides for immunisation has the disadvantage that protein folding to produce accurate secondary and tertiary structures is unlikely to occur as the peptides are too short (Xing *et al.*, 1992). One means of overcoming these problems is to express a number of the repetitive units in a bacterial expression system, as this would provide non-glycosylated polypeptide of sufficient length to fold in a manner similar to the natural mucin. Using this approach, we now report two Mabs which recognise peptide epitopes on breast cancer mucin. One such Mab, VA1, reacted with breast cancer and normal tissue, whereas a second Mab VA2, showed a high degree of specificity for breast cancer tissue.

Correspondence: I.F.C. McKenzie.
Received 29 June 1992; and in revised form 18 November 1992.

### Materials and methods

### *Production of soluble GST-MUC1 fusion protein*

A 309 base pair insert (PDF9.3) encoding a little more than five repeats of a 60 base pair motif from the VNTR region of MUC1 cDNA (Siddiqui *et al.*, 1988) was subcloned into the bacterial expression vector pGEX-3X, in the correct reading frame and orientation (Smith & Johnson, 1988). Fusion protein, consisting of glutathione-S-transferase (GST) and MUC1 VNTR, was induced with 0.1 mM IPTG (Smith & Johnson, 1988). Cells were collected by centrifugation, washed and lysed by sonication in buffer containing 1% (v/v) Triton X-100. Supernatant containing the soluble fusion protein was mixed with glutathione-agarose beads (sulphur-linked) (Sigma, St. Louis, USA) and collected by centrifugation. The fusion protein was eluted with buffer containing 5 mM reduced glutathione, dialysed against phosphate buffered saline and analysed by SDS-PAGE.

### *Preparation of peptides and HMFG*

Peptides (Table I) were synthesised using an Applied Biosystems Model 430A automated peptide synthesiser (Hodges & Merrifield, 1975; Kent & Hood, 1984). HMFG and deglycosylated HMFG were prepared as previously described (Xing *et al.*, 1989).

### *Immunisation and production of Mabs*

(CBA × BALB/c)F1 mice (females aged 8 weeks) were immunised intraperitoneally with 50 µg of the fusion protein emulsified with complete Freund's adjuvant and this was repeated four and six weeks later. Three days prior to cell fusion a boost of soluble fusion protein was given intravenously and a cell fusion performed (Thompson *et al.*, 1983). Hybridomas were selected on the basis of a strong reaction of the supernatant of the immunogen, and a negative reaction with an irrelevant synthetic peptide (T4N1) and GST. The immunoglobulin isotype of the Mabs was determined by Ouchterlony gel immunodiffusion using rabbit antisera to mouse immunoglobulins (Serotec, Oxford, England). The hybridomas were grown as ascites tumours in pristane-primed (CBA × BALB/c)F1 mice and the Mabs (IgG1 subclass) were purified from the mouse ascites fluid using a Protein A-Sepharose 4B column.

### *Immunoperoxidase staining and ELISA assays*

The immunoperoxidase staining on tissue sections was performed (Stacker *et al.*, 1985) and the reactions graded ac-

**Table I** Reaction of monoclonal antibodies with synthetic peptides tested by solid phase ELISA

| *Peptide amino acid composition (No.)* | | *Optical density VA1* | *VA2* |
|---|---|---|---|
| VNTR-GST | (PDTRPAPGSTAPPAHGVTSA) x 5-GST (103) | 2.00 | 1.90 |
| P1-24 | PDTRPAPGSTAPPAHGVTSAPDTR (24) | 0.48 | 0.11 |
| P1-15 | PDTRPAPGSTAPPAH (15) | 0.28 | 0.09 |
| P5-20 | PAPGSTAPPAHGVTSA (16) | 0.08 | 0.08 |
| P13-32 | PAHGVTSAPDTRPAPGSTAP (20) | 0.12 | 0.05 |
| C-P13-32 | C-PAHGVTSAPDTRPAPGSTAP (21) | 1.99 | 1.83 |
| T4N1 | KTLVLGLEQESAELPCEY (19) | 0.11 | 0.13 |
| HMFG | | 0.39 | 0.90 |
| D.HMFG | | 1.26 | 1.25 |
| GST | | 0.22 | 0.17 |

Optical Density values taken at one-tenth dilution of tissue culture supernatant of hybridomas. HMFG = Human milk fat globule membrane. D.HMFG = deglycosylated HMFG.

cording to the percentage of cells stained: 0% ( − ), <25% (1 + ), 25–50% (2 + ), 50–75% (3 + ),>75% (4 + ) and the intensity of staining: negative ( − ), weak (1 + ), moderate (2 + ), strong (3 + ), very strong (4 + ). The solid phase and the inhibition (liquid phase) ELISA tests were performed as described (Xing *et al.*, 1989; Xing *et al.*, 1991). For the direct test, 20 $\mu g\ ml^{-1}$ of peptides and 10 $\mu g\ ml^{-1}$ of HMFG and deglycosylated HMFG were coated in the wells of a microtitre plate. Non specific binding was blocked and the antibody reacted for 2 h. For the inhibition studies C-p13-32 pre-coated plates (20 $\mu g\ ml^{-1}$) were used to test the peptides, which at increasing concentrations ($3.6 \times 10^{-5}$–0.08 mM) were mixed with the antibodies and left on the plates for 2 h.

*Epitope mapping using the pepscan method*

Peptides corresponding to the MUC1 sequence were synthesised on polyethylene pins and consisted of twenty overlapping 6-mer peptides e.g. PDTRPA, DTRPAP, TRPAPG ....., APDTRP. The Mabs were tested for binding to the peptides on pins using the ELISA method (Xing *et al.*, 1991).

### Results

*Production of Mabs detecting a GST-MUC1 fusion protein*

A bacterial fusion protein consisting of GST (26 Kd) and five repeats of the MUC1 VNTR 20-mer peptide was induced in *E. coli*, purified and used to raise Mabs. Hybridomas were selected on the basis of reactivity with the MUC1 fusion protein, lack of reactivity with an irrelevant synthetic peptide (T4N1) and GST (Table I), and a reaction on formalin fixed breast cancer tissues detected by the immunoperoxidase technique. Two hybridomas, VA1 and VA2 were selected for further study on the basis of the selection criteria.

*Reaction of Mabs with synthetic peptides, HMFG and the fusion protein*

Each antibody was tested on natural and deglycosylated HMFG, the fusion protein and on a number of peptides whose sequence was based on that of the MUC1 VNTR sequence (Table I). In addition to the immunising fusion protein (VNTR-GST), VA1 reacted strongly with C-p13-32 (a dimeric form of p13-32, composed of disulphide linked monomers) and with deglycosylated HMFG but weakly with the p13-32 monomer peptide and with the fully glycosylated HMFG. The reactivity with the dimer peptide (C-p13-32) but not with p13-32 or other monomeric peptides (Table I) was of interest and indicated that the antibodies may be more reactive when a secondary structure is allowed to form. We found however that small peptides synthesised on pins were reactive (see below). To further study the reactions, the peptides were used in inhibition studies (Figure 1a, b) and the differences noted in direct studies were not observed. For these studies, peptides in solution were used to inhibit the reaction of VA1 (Figure 1a) with C-p13-32 on the plate. The peptides C-p13-32, p13-32, p1-15, p1-24 and p5-20 all inhibited; in particular, C-p13-32 and p13-32 on a molar basis, gave similar inhibition. HMFG also gave some inhibition, although virtually no reaction was noted when HMFG was coated on the plate (Table I). The reactions of VA2 were similar to VA1 but with some notable differences. In addition to the immunisating fusion protein, VA2 reacted with both forms of HMFG. The peptides C-p13-32, p13-32, p1-15, and p1-24 all inhibited in the inhibition assay (Figure 1b) although in the solid phase ELISA only C-p13-32 was reactive (Table I).

*Reaction of Mabs with human tissue*

The reactivities of the two antibodies VA1 and VA2 with various tumours are shown in Table II and Table IV. Using the immunoperoxidase staining technique Mab VA1 reacted with 46/53 (87%) breast cancers and VA2 with 48/53 (91%) (Figure 2). VA1 and VA2 were clearly not breast cancer specific as they reacted with other cancers (Tables II and IV). VA1 gave weak reactions with formalin fixed normal breast epithelial cells in acini and ducts, but a stronger reaction on fresh breast tissues (Figure 3a), whereas VA2 showed no such reaction on all formalin fixed tissues and a weak reaction with fresh normal breasts (Figure 3b). Both Mabs gave additional weak reactions (luminal, cell surface, secretion, cytoplasmic) with other normal tissues (Table III and Table V). Both VA1 and VA2 reacted strongly with normal colon tissues (all cells within the gland were positive). MUC1 protein is not thought to be expressed by normal colon but we note that normal colon does indeed express MUC1 (Figure 4). Likewise with normal ovary both antibodies reacted with formalin fixed tissues. In general, the staining was weaker on normal tissues than on cancer tissues and stronger on fresh tissue than on formalin fixed tissue. Comparing the two Mabs with Mab BC2 (an anti-MUC1 antibody produced using whole mucin HMFG as the immunogen and which reacted with the amino acids APDTR) some differences were noted. The staining intensity of BC2 on both normal kidney and carcinoma of the kidney was intense, in contrast to VA1 and VA2 where much weaker reactions were observed. In addition the staining intensity of VA1 and VA2 in some breast carcinomas was more intense than that of BC2. Also the staining pattern of BC2 on colon carcinoma (formalin fixed) was different to that of VA1 and VA2 as BC2 showed a luminal staining pattern and stained the secretions, whereas VA1 and VA2 showed only a weak cytoplasmic staining only (data not shown). Thus, the staining properties of the anti-

**Figure 1** Inhibition assay of the binding of Mab (a) VA1 and (b) VA2 to C-p13-32 using synthetic peptides at increasing concentrations ($3.6 \times 10^{-5}$–0.08 mM).

**Table II** Reaction of Mabs VA1 and VA2 with formalin fixed human tumours by immunoperoxidase staining

| *Tumour tissue* | *VA1* No. of +ve staining/total tested | *VA1* % of cancer cells staining[a] | | | | *VA1* staining intensity[b] | | | | *VA2* No. of +ve staining/total tested | *VA2* % of cancer cells staining | | | | *VA2* staining intensity | | | |
|---|---|---|---|---|---|---|---|---|---|---|---|---|---|---|---|---|---|---|
| | | + | 2+ | 3+ | 4+ | + | 2+ | 3+ | 4+ | | + | 2+ | 3+ | 4+ | + | 2+ | 3+ | 4+ |
| Breast (Br.) | 46/53 | 17 | 8 | 10 | 11 | 5 | 14 | 14 | 13 | 48/53 | 23 | 7 | 5 | 12 | 11 | 11 | 10 | 16 |
| Ovary | 3/5 | 1 | 0 | 0 | 2 | 1 | 1 | 0 | 1 | 2/5 | 0 | 0 | 1 | 1 | 1 | 1 | 0 | 0 |
| Pancreas | 2/2 | 0 | 1 | 1 | 0 | 0 | 0 | 1 | 1 | 2/2 | 0 | 1 | 0 | 1 | 0 | 0 | 2 | 0 |
| Kidney | 8/12 | 3 | 3 | 2 | 0 | 4 | 1 | 1 | 2 | 7/12 | 3 | 2 | 1 | 1 | 1 | 2 | 2 | 2 |
| Prostate | 1/2 | 0 | 1 | 0 | 0 | 0 | 1 | 0 | 0 | 2/2 | 2 | 0 | 0 | 0 | 1 | 1 | 0 | 0 |
| Stomach | 5/5 | 2 | 0 | 1 | 2 | 0 | 3 | 0 | 2 | 5/5 | 2 | 0 | 1 | 2 | 1 | 2 | 0 | 2 |
| Liver | 1/1 | 0 | 1 | 0 | 0 | 0 | 0 | 1 | 0 | 1/1 | 1 | 0 | 0 | 0 | 0 | 0 | 1 | 0 |
| Lung | 7/12 | 3 | 4 | 0 | 0 | 1 | 4 | 2 | 0 | 6/12 | 3 | 2 | 1 | 0 | 0 | 5 | 0 | 1 |
| Colon | 9/22 | 6 | 2 | 1 | 0 | 3 | 3 | 2 | 1 | 5/22 | 4 | 1 | 0 | 0 | 1 | 3 | 1 | 0 |
| Br. fibroadenoma | 3/6 | 1 | 0 | 1 | 1 | 0 | 2 | 1 | 0 | 3/6 | 2 | 1 | 0 | 0 | 1 | 2 | 0 | 0 |
| Br. Cystic hyperplasia | 0/4 | 0 | 0 | 0 | 0 | 0 | 0 | 0 | 0 | 0/4 | 0 | 0 | 0 | 0 | 0 | 0 | 0 | 0 |

[a]Staining was graded: 0% (−); <25% (+); 25–50% (2+); 50–75% (3+); >75% (4+); [b]Staining intensity was graded: negative (−); moderate (2+); strong (3+); very strong (4+); The gradings of [a] and [b] were performed by two individuals.

**Table III** Reaction of Mabs VA1 and VA2 with formalin fixed normal tissue by immunoperoxidase staining

| *Tissue* | *VA1* No. of +ve staining/total tested | *VA1* % of normal cells staining[a] | | | | *VA1* staining intensity[b] | | | | *VA2* No. of +ve staining/total tested | *VA2* % of normal cells staining | | | | *VA2* staining intensity | | | |
|---|---|---|---|---|---|---|---|---|---|---|---|---|---|---|---|---|---|---|
| | | + | 2+ | 3+ | 4+ | + | 2+ | 3+ | 4+ | | + | 2+ | 3+ | 4+ | + | 2+ | 3+ | 4+ |
| Breast | 5/10 | 3 | 2 | 0 | 0 | 1 | 4 | 0 | 0 | 0/10 | 0 | 0 | 0 | 0 | 0 | 0 | 0 | 0 |
| Colon | 6/13 | 2 | 1 | 1 | 2 | 2 | 1 | 2 | 1 | 4/13 | 1 | 2 | 0 | 1 | 2 | 1 | 1 | 0 |
| Ovary | 2/2 | 1 | 0 | 1 | 0 | 0 | 2 | 0 | 0 | 2/2 | 1 | 0 | 1 | 0 | 0 | 2 | 0 | 0 |
| Salivary gland | 2/2 | 0 | 0 | 2 | 0 | 0 | 0 | 2 | 0 | 2/2 | 0 | 0 | 2 | 0 | 0 | 1 | 1 | 0 |
| Pancreas | 2/2 | 0 | 0 | 0 | 2 | 0 | 0 | 0 | 2 | 2/2 | 0 | 0 | 0 | 2 | 0 | 0 | 0 | 2 |
| Small intestine | 1/1 | 0 | 1 | 0 | 0 | 0 | 1 | 0 | 0 | 1/1 | 1 | 0 | 0 | 0 | 1 | 0 | 0 | 0 |
| Cervix | 0/1 | 0 | 0 | 0 | 0 | 0 | 0 | 0 | 0 | 0/1 | 0 | 0 | 0 | 0 | 0 | 0 | 0 | 0 |
| Gall bladder | 1/4 | 1 | 0 | 0 | 0 | 1 | 0 | 0 | 0 | 0/4 | 0 | 0 | 0 | 0 | 0 | 0 | 0 | 0 |
| Stomach | 2/2 | 0 | 0 | 1 | 1 | 0 | 0 | 1 | 1 | 2/2 | 0 | 0 | 1 | 1 | 0 | 0 | 1 | 1 |
| Liver | 0/1 | 0 | 0 | 0 | 0 | 0 | 0 | 0 | 0 | 0/1 | 0 | 0 | 0 | 0 | 0 | 0 | 0 | 0 |
| Lung | 3/6 | 0 | 3 | 0 | 0 | 1 | 2 | 0 | 0 | 5/6 | 2 | 3 | 0 | 0 | 2 | 3 | 0 | 0 |
| Kidney | 3/4 | 2 | 0 | 1 | 0 | 0 | 2 | 1 | 0 | 3/4 | 1 | 1 | 1 | 0 | 1 | 0 | 2 | 0 |

[a]Staining was graded: 0% (−); <25% (+); 25–50% (2+); 50–75% (3+); >75% (4+); [b]Staining intensity was graded: negative (−); weak (+) moderate (2+); strong (3+); very strong (4+); The gradings of [a] and [b] were performed by two individuals.

**Figure 2** Immunoperoxidase staining of infiltrating duct breast carcinoma tissue with antibody VA2 showing intense cell surface and cytoplasmic staining (original manification × 20). VA1 showed similar staining.

**Table IV** Reaction of Mabs VA1 and VA2 with fresh human tumour tissues by immunoperoxidase staining

| | *VA1* | | | | | | | | | *VA2* | | | | | | | | |
|---|---|---|---|---|---|---|---|---|---|---|---|---|---|---|---|---|---|---|
| *Tumour tissue* | *No. of +ve staining/ total tested* | *% of cancer cells staining*[a] | | | | *staining intensity*[b] | | | | *No. of +ve staining/ total tested* | *% of cancer cells staining* | | | | *staining intensity* | | | |
| | | + | 2+ | 3+ | 4+ | + | 2+ | 3+ | 4+ | | + | 2+ | 3+ | 4+ | + | 2+ | 3+ | 4+ |
| Breast | 5/5 | 0 | 2 | 0 | 3 | 0 | 1 | 3 | 1 | 5/5 | 0 | 2 | 0 | 3 | 0 | 2 | 2 | 1 |
| Ovary | 1/1 | 0 | 0 | 1 | 0 | 0 | 0 | 1 | 0 | 1/1 | 0 | 0 | 0 | 1 | 0 | 1 | 0 | 0 |
| Stomach | 3/3 | 0 | 1 | 0 | 2 | 0 | 0 | 1 | 2 | 3/3 | 0 | 1 | 0 | 2 | 0 | 0 | 1 | 2 |
| Colon | 4/5 | 1 | 2 | 1 | 0 | 0 | 2 | 1 | 1 | 4/5 | 2 | 1 | 1 | 0 | 2 | 0 | 1 | 1 |
| Lung | 10/10 | 3 | 2 | 2 | 3 | 2 | 3 | 3 | 2 | 10/10 | 4 | 2 | 1 | 3 | 3 | 3 | 2 | 2 |

[a]Staining was graded: 0% (−); <25% (+); 25–50% (2+); 50–75% (3+); >75% (4+); [b]Staining intensity was graded: negative (−); weak (+) moderate (2+); strong (3+); very strong (4+); The gradings of [a] and [b] were performed by two individuals.

fusion protein antibodies VA1 and VA2 differ substantially from those of BC2.

### *Epitopes detected by VA1 and VA2*

To define the precise epitopes detected by VA1 and VA2, the 'pepscan' method was used. In this method overlapping peptides were examined and the peptide RPAPGS showed a strong reaction with VA1 and the peptides TRPAPG, PAPGST and APGSTA gave a weaker reaction. APG is common and is a partial epitope whereas RPAPGS the full epitope (Figure 5a). For VA2 the two peptides DTRPAP and PDTRPA showed strong reactivity and the common amino acids in the epitope are DTRPA (Figure 5b).

**Figure 3** Immunoperoxidase staining of normal breast tissue with (**a**) antibody VA1 and (**b**) antibody VA2 showing the amount of staining (original magnification × 20).

**Table V** Reaction of Mabs VA1 and VA2 with normal fresh tissues by immunoperoxidase staining

| | *VA1* | | | | | | | | | *VA2* | | | | | | | | |
|---|---|---|---|---|---|---|---|---|---|---|---|---|---|---|---|---|---|---|
| *Tissue* | *No. of +ve staining/ total tested* | *% of normal cells staining*[a] | | | | *staining intensity*[b] | | | | *No. of +ve staining/ total tested* | *% of normal cells staining* | | | | *staining intensity* | | | |
| | | + | 2+ | 3+ | 4+ | + | 2+ | 3+ | 4+ | | + | 2+ | 3+ | 4+ | + | 2+ | 3+ | 4+ |
| Breast | 3/3 | 0 | 0 | 1 | 2 | 0 | 0 | 1 | 2 | 2/3 | 2 | 0 | 0 | 0 | 1 | 1 | 0 | 0 |
| Trachea | 1/2 | 0 | 1 | 0 | 0 | 0 | 1 | 0 | 0 | 1/2 | 0 | 1 | 0 | 0 | 0 | 1 | 0 | 0 |
| Lung | 5/5 | 0 | 1 | 3 | 1 | 1 | 2 | 1 | 1 | 5/5 | 0 | 3 | 1 | 1 | 1 | 3 | 0 | 1 |
| Salivary gland | 1/1 | 0 | 0 | 0 | 1 | 0 | 0 | 0 | 1 | 1/1 | 0 | 0 | 1 | 0 | 0 | 0 | 1 | 0 |
| Stomach | 4/5 | 0 | 1 | 0 | 3 | 0 | 1 | 2 | 1 | 4/5 | 1 | 0 | 2 | 1 | 1 | 2 | 0 | 1 |
| Small intestine | 3/3 | 0 | 1 | 1 | 1 | 0 | 2 | 0 | 1 | 3/3 | 0 | 1 | 1 | 1 | 1 | 1 | 1 | 0 |
| Colon | 8/8 | 0 | 0 | 1 | 7 | 0 | 0 | 0 | 8 | 8/8 | 0 | 1 | 1 | 6 | 0 | 0 | 5 | 3 |
| Rectum | 4/4 | 0 | 0 | 2 | 2 | 0 | 1 | 1 | 2 | 4/4 | 0 | 2 | 0 | 2 | 0 | 2 | 1 | 1 |
| Pancreas | 3/3 | 0 | 0 | 2 | 1 | 0 | 1 | 2 | 1 | 3/3 | 0 | 0 | 2 | 1 | 0 | 0 | 1 | 2 |
| Appendix | 0/1 | 0 | 0 | 0 | 0 | 0 | 0 | 0 | 0 | 0/1 | 0 | 0 | 0 | 0 | 0 | 0 | 0 | 0 |
| Spleen | 1/1 | 0 | 0 | 0 | 1 | 0 | 0 | 0 | 1 | 1/1 | 0 | 0 | 0 | 1 | 0 | 0 | 0 | 1 |
| Kidney | 3/3 | 0 | 0 | 3 | 0 | 0 | 1 | 2 | 0 | 3/3 | 0 | 1 | 2 | 0 | 0 | 0 | 2 | 1 |
| Liver | 0/2 | 0 | 0 | 0 | 0 | 0 | 0 | 0 | 0 | 0/2 | 0 | 0 | 0 | 0 | 0 | 0 | 0 | 0 |
| Bladder | 0/3 | 0 | 0 | 0 | 0 | 0 | 0 | 0 | 0 | 0/3 | 0 | 0 | 0 | 0 | 0 | 0 | 0 | 0 |
| Gall bladder | 0/3 | 0 | 0 | 0 | 0 | 0 | 0 | 0 | 0 | 0/3 | 0 | 0 | 0 | 0 | 0 | 0 | 0 | 0 |
| Ovary | 0/1 | 0 | 0 | 0 | 0 | 0 | 0 | 0 | 0 | 0/1 | 0 | 0 | 0 | 0 | 0 | 0 | 0 | 0 |
| Uterus | 0/1 | 0 | 0 | 0 | 0 | 0 | 0 | 0 | 0 | 0/1 | 0 | 0 | 0 | 0 | 0 | 0 | 0 | 0 |

[a]Staining was graded: 0% (−); <25% (+); 25–50% (2+); 50–75% (3+); >75% (4+); [b]Staining intensity was graded: negative (−); weak (+) moderate (2+); strong (3+); very strong (4+); The gradings of [a] and [b] were performed by two individuals.

**Figure 4** Immunoperoxidase staining of colon carcinoma tissue stained with antibody VA1 showing intense glandular staining (original magnification × 20). VA2 showed similar staining.

### Discussion

This report describes the production of Mabs VA1 and VA2 against a MUC1 bacterial fusion protein. A fusion protein derived from the MUC1 cDNA clone which contained five VNTR's of 100 amino acids in length (which is too long to be realistically produced as a synthetic peptide) was produced in *E. coli*, affinity purified and used to generate two Mabs, VA1 and VA2. These antibodies have unique characteristics when compared to Mabs made to natural mucin (BC2). Antibody VA1 reacted with most breast carcinoma tissues and showed a weak reaction with normal breast tissues. It also reacted with a variety of other epithelial tissues, both cancer and normal tissue. By contrast Mab VA2 reacted with breast carcinomas but was non-reactive on all ten formalin fixed normal breast tissues and a weak reaction with fresh breast tissues. This clear differentiation between normal and malignant breast tissues makes both antibodies potentially good candidates for clinical uses, particularly VA2. Comparing Mabs VA1 and VA2 with the anti-mucin Mab BC2, different staining patterns were noted, even though BC2 also reacts with MUC1, but with a different peptide (APDTR).

Mab NCRC-11 (Price *et al.*, 1990) recognises the epitope RPA. This epitope is in the hydrophilic turn region of the peptide and suggests that the peptide core is exterior of the glycoprotein where the antibody binds. The amino acids PDTRPAP are exposed in cancer mucins, and indeed VA1 and VA2 have reactive epitopes within this region. It was also shown that in the 20 amino acid repeat there is no potential for β sheet or α helix formation (Price *et al.*, 1990). High field NMR studies were taken from 11 amino acid fragment of the sequence, and revealing elements of secondary structure to be present (Tendler *et al.*, 1990). A type 1 β turn from D(2)-R(4) was found which extends by P(3) being in the trans form. The turn region extends into the epitopes known for Mabs such as C595 and NCR-11 (Price *et al.*, 1990) as well as VA1 and VA2.

VA1 and VA2 gave differential reactions on tissue sections, HMFG, deglycosylated HMFG and on peptides. Firstly VA1 reacted with both cancer and normal tissues, not with HMFG but with deglycosylated HMFG and with the peptide epitope RPAPGS. The pattern of reactivity of VA1 fits what is now considered to occur with MUC1 expression in normal and malignant tissue. It has been proposed, largely on the

**Figure 5** Reactivity of 1/10 dilution of tissue culture supernatant of (a) VA1 and (b) VA2 with synthetic peptides synthesised on pins.

basis of the reactivity of the SM3 antibody (made to deglycosylated HMFG) (Burchell *et al.*, 1987) that there is altered or defective glycosylation in malignancy, so antibodies to MUC1 core proteins react weakly with normal tissue and HMFG, but more strongly on deglycosylated HMFG and cancer cells, i.e. VA1 pattern. The reactivity of VA2 is similar other than for the stronger reaction on whole HMFG. The reason for this is not clear, possibly the DTRPA epitope is exposed in HMFG than the epitope RPAPGS; it is possible that threonine (T) in DTRPA is not glycosylated, clearly making this epitope more accessible. Other DTR reactive Abs are SM3 and HMFG (Burchell *et al.*, 1989) and SM3 in particular does not react with HMFG but does so with deglycosylated HMFG.

Both antibodies showed no reaction with synthetic peptides p1-15, p1-24, and p13-32 in a solid phase ELISA (Table I) even though they contain the epitope recognised by the antibodies. However both antibodies could react with the peptides in liquid phase (in the inhibition studies). It is likely that the peptides in a solid phase lose conformation as they attach to the plate, and the antibodies are unable to see their corresponding determinants. Thus, although the predominant reaction is with the primary sequence of the peptide, some conformational alteration occurs in solution or when the peptide is attached at one end, as with the pins, permitting stronger reaction with the antibody.

Fusion proteins have recently been used to test antibodies, e.g. MUC1 for HMFG-1 and HMFG-2 and for anti-CEA antibodies to map the domain reactivity (Burchell *et al.*, 1987; Hass *et al.*, 1991). We now show that fusion proteins can also be used to produce antibodies. Are these of any advantage over HMFG, tumour extracts or synthetic peptide – it remains to be seen in various diagnostic and therapeutic tests. However the differential reactivity on HMFG and deglycosylated HMFG, on C-p13-32 *vs* p13-32 and on tumour and normal tissues (i.e. VA2) indicate that these antibodies may be the most specific made thus far. The Mab SM3 (Burchell *et al.*, 1987) which recognises an epitope exposed in the mucin as processed by the tumour cells and not exposed on normal mucin, gave weak reactions with normal breast and other normal tissues. VA2 reacted with normal tissues and reacted weakly with only fresh normal breast, indicating that it may be a useful diagnostic or therapeutic agent for breast cancer.

We would like to thank Dr G.A. Pietersz for helpful discussions.

### References

BOLAND, C.R. & DESHMUCK, G.D. (1990). The carbohydrate composition of mucin in colonic cancer. *Gastroenterology*, **98**, 1170-1177.

BURCHELL, J., GENDLER, S., PAPADIMITRIOU, J.T., GIRLING, A., LEWIS, A., MILLIS, R. & LAMPORT, D. (1987). Development and characterization of breast cancer reactive monoclonal antibodies directed to the core protein of the human milk mucin. *Cancer Res.*, **47**, 5476-5482.

BURCHELL, J., PAPADIMITRIOU, J.T., BOSHELL, M., GENDLER, S. & DUHIG, T. (1989). A short sequence within the amino acid tandem repeat of a cancer-associated mucin, contains immunodominant epitopes. *Int. J. Cancer*, **44**, 691-696.

CROCKER, G. & PRICE, M.R. (1987). Genetic polymorphism of high molecular weight glycoproteins: a comparative study in normal individuals and breast cancer patients. *Br. J. Cancer*, **55**, 651-652.

GENDLER, S., PAPADIMITRIOU, J.T., DUHIG, T., ROTHBARD, J. & BURCHELL, J. (1988). A highly immunogenic region of a human polymorphic epithelial mucin expressed by carcinomas is made up of tandem repeats. *J. Biol. Chem.*, **263**, 12820-12823.

HARISCH, F.G. & UHLENBRUCK, G. (1989). Structures of neutral O-linked polylactosaminoglycans on human skim milk mucins. *J. Biol. Chem.*, **264**, 872-883.

HASS, G.M., BOLLING, T.J., KINDERS, R.J., HENSLEE, J.G., MANDECKI, W., DORWIN, S.A. & SHIVELY, J.E. (1991). Preparation of synthetic polypeptide domains of carcinoembryonic antigen and their use in epitope mapping. *Cancer Res.*, **51**, 1876-1882.

HODGES, R.S. & MERRIFIELD, R.B. (1975). Monitoring of solid phase peptide synthesis by an automated spectrophotometric picrate method. *Anal. Biochem.*, **65**, 241-272.

KENT, S.B.H. & HOOD, L.E.A. (1984). A novel approach to automated peptide synthesis based on new insights into solid phase chemistry. In *Peptides Chemistry*, Izumiya, N. (ed) pp. 217–222. Protein Research Foundation: Osaka, Japan.

MARJOLIJN, J.L., LIGTENBERG, M.J.L., VOS, H.L., ANNEMIEK, M.C., GENNISSEN, A.M.C. & HILKENS, J. (1990). Episialin, a carcinoma-associated mucin is generated by a polymorphic gene encoding splice variants with alternating amino termini. *J. Biol. Chem.*, **265**, 5573–5578.

PRICE, M.R., HUDECZ, F., O'SULLIVAN, C., BALDWIN, R.W., EDWARDS, P.M. & TENDLER, S.J.B. (1990). Immunological and structural features of the protein core of human polymorphic epithelial mucin. *Molec. Immunol.*, **27**, 795–802.

SIDDIQUI, J., ABE, M., HAYES, D., SHANI, E., YUNIS, E. & KUFE, D. (1988). Isolation and sequencing of a cDNA coding for the human DF3 breast carcinoma-associated antigen. *Proc. Natl Acad. Sci.*, **85**, 2320–2323.

SMITH, D.B. & JOHNSON, K.S. (1988). Single-step purification of polypeptides expressed in Escherichia coli as fusions with glutathione S-transferase. *Gene.*, **67**, 31–40.

STACKER, S.A., THOMPSON, C.H., RIGLAR, C. & MCKENZIE, I.F.C. (1985). A new breast carcinoma antigen defined by a monoclonal antibody. *J. Natl Cancer Inst.*, **75**, 801–811.

TENDLER, S.J.B. (1990). Elements of secondary structure in a human epithelial mucin core peptide fragment. *J. Biochem.*, **267**, 733–737.

THOMPSON, C.H., JONES, S.L., WHITEHEAD, R.H. & MCKENZIE, I.F.C. (1983). A human breast tissue-associated antigen detected by a monoclonal antibody. *J. Natl Cancer Inst.*, **70**, 409–419.

XING, P.X., TJANDRA, J.J., STACKER, S.A., TEH, J.G., THOMPSON, C.H., MCLAUGHLIN, P.J. & MCKENZIE, I.F.C. (1989). Monoclonal antibodies reactive with mucin expressed in breast cancer. *Immunol. Cell Biol.*, **67**, 183–195.

XING, P.X., REYNOLDS, K., PIETERSZ, G.A. & MCKENZIE, I.F.C. (1991). Effect of variation in peptide sequence on anti-human milk fat globule membrane antibody reactions. *Immunol.*, **72**, 304–311.

XING, P.X., PRENZOSKA, J., QUELCH, K. & MCKENZIE, I.F.C. (1992). Second generation anti-MUC1 peptide monoclonal antibodies. *Cancer Res.*, **52**, 2310–2317.